# The Functional Origin of Oral Word Production Deficits in the Logopenic Variant of Primary Progressive Aphasia: A Systematic Review

**DOI:** 10.3390/brainsci15020111

**Published:** 2025-01-24

**Authors:** Amra Hasanovic, Joël Macoir, Amélie Sanfaçon-Verret, Laura Monetta

**Affiliations:** 1 École des Sciences de la Réadaptation, Faculté de Médecine, Université Laval, Québec, QC G1V 0A6, Canada; amra.hasanovic.1@ulaval.ca (A.H.); amelie.sanfacon-verret.1@ulaval.ca (A.S.-V.); laura.monetta@fmed.ulaval.ca (L.M.); 2 Centre Interdisciplinaire de Recherche en Réadaptation et Intégration Sociale (CIRRIS), Québec, QC G1M 2S8, Canada; 3 Centre de Recherche CERVO, Québec, QC G1J 2G3, Canada

**Keywords:** language, communication disorders, primary progressive aphasia

## Abstract

Background/Objectives: Oral word production (OWP) deficits are prominent in the logopenic variant of primary progressive aphasia (lvPPA); however, their functional origin remains unclear. Some studies suggest a lexical, post-lexical, or even a combined functional origin of these deficits. The aim of the present study was to synthesize and analyze the information on the functional origin of the OWP deficits in patients with lvPPA. Methods: A quantitative systematic literature review was carried out using four databases: CINAHL, PsycINFO, Linguistics and Language Behavior Abstracts, and PubMed. Fourteen studies, including a total of 243 patients with lvPPA, and reporting results on picture naming and/or word and/or pseudoword repetition, were selected. Results: The overall findings of this review highlighted that two main functional origins appear to explain the OWP deficits in lvPPA: a lexical impairment affecting lexical processing and a post-lexical impairment affecting phonological short-term memory. Interestingly, the possibility of a third functional origin, affecting the semantic processing level, was also suggested by some studies. Conclusions: We concluded that the presence of different functional origins of OWP in this population may be explained, at least partially, by the diversity of assessment tasks used in studies and the varied manipulation and control of psycholinguistic properties of words (e.g., frequency, length), as well as the various interpretations and analyses of the participants’ errors. Further studies are needed to substantiate these findings by examining all the components involved in OWP, carefully manipulating the psycholinguistic properties and qualitatively analyzing the errors made by lvPPA participants.

## 1. Introduction

Primary progressive aphasia (PPA) is a neurodegenerative condition characterized by a progressive deterioration of language during the first two years of the disorder, impacting receptive and/or expressive skills [1]. Three main variants of PPA have been characterized: semantic (svPPA), agrammatic/non-fluent (nfvAPP), and logopenic (lvPPA) [2]. The variant of interest in the present study is lvPPA. The core features of lvPPA are the presence of anomia in spontaneous speech and picture naming, and a deficit in sentence repetition. Additionally, at least three of the following features must be present to diagnose lvPPA: production of phonological errors in spontaneous speech and picture naming, preservation of object knowledge and single-word comprehension, and/or absence of agrammatism [2]. The inclusion of the preservation of object knowledge and single-word comprehension as an optional criterion may suggest that semantic processing is relatively unimpaired in lvPPA, even though its preservation is not directly mentioned in the criteria [3]. From a neuroanatomical point of view, patients with lvPPA are known to present with cerebral atrophy in the left inferior parietal lobe and the left posterior temporal lobe [4]. The atrophy of these cerebral regions is known to be the origin of the language deficits seen in the lvPPA population [5]. Oral word production (OWP) is a complex process that requires the activation of various interrelated cognitive components [6,7], namely the semantic memory, the phonological lexicon, the phonological short-term memory, and the articulatory system [7]. OWP impairments are one of the core features of lvPPA, but their functional origin is still debated as they may be the result of underlying deficits associated with any of those components [1,4]. Some studies have attributed the functional origin of OWP deficits to an impairment localized at the lexical level (lexical anomia), more specifically to the access to phonological representations (e.g., [8]). Other studies have suggested that the deficits may rather reflect a reduced capacity of the phonological short-term memory (e.g., [9]).

Different factors can play a role in the apparent lack of consensus between studies regarding the functional origin of OWP impairments in lvPPA. The heterogeneity of the tasks employed by researchers to assess OWP in the lvPPA population [10] can complicate comparisons between participants across different studies. Indeed, OWP can typically be assessed through a variety of tasks, such as word repetition, word reading, or picture naming [11]. Additionally, a rigorous qualitative analysis of the errors produced by participants [12] is not always conducted by researchers. The same applies to the manipulation and control of the psycholinguistic properties of the stimuli used in the tasks [13], which are known to influence the speed and accuracy of word production [14]. All this variability inevitably contributes to challenges in determining the functional origin of OWP deficits in lvPPA.

Identifying the functional origin of OWP deficits will enhance our understanding of lvPPA and contribute to the development of targeted interventions that directly address the functional origin of the difficulties. The objective of this quantitative systematic review was to synthesize and analyze the current literature on the functional origins of OWP deficits in lvPPA. 

## 2. Materials and Methods

### 2.1. Search Strategy

A quantitative systematic review on the functional origin of OWP deficits in the lvPPA was conducted following the Preferred Reporting Items for Systematic Reviews and Meta-Analyses (PRISMA) guidelines [15], consisting of (1) the formulation of a review objective; (2) the definition of eligibility criteria, (3) the conduction of a search of the scientific literature, and (4) a study selection based on titles/abstracts and then on full texts. The systematic search was conducted across four electronic databases (MedLine, PsycINFO, Linguistics and Language Behavior Abstracts, CINHAL) in August 2024. All the selected databases list articles in the health sciences or linguistics domains. The search terms included free vocabulary (i.e., text keywords in the title or abstract) related to two main keywords, logopenic primary progressive aphasia, and anomia. The following free vocabulary was used in each database: “Primary progressive aphasia” or “PPA” or “logopenic” or “lv-PPA” or “PPA-L” AND anomi* or lexical retriev* or word retriev* or “word production” or “word finding” or “naming” or “repetition”. The search included controlled vocabulary (i.e., index terms from the thesaurus of each database) specific to each database as well. The complete search terminology can be found in the Supplementary Materials. Although it involves OWP processes, reading words aloud was not included in the search, as this skill also involves different cognitive processes related to written information.

### 2.2. Study Selection and Data Extraction

Covidence, a software program designed for systematic reviews, was used in the following four different phases of the study selection: (1) identification of the articles in the databases and automatic removal of duplicates, (2) screening of the articles using titles and abstracts, (3) eligibility of the articles after full-text reading, and (4) inclusion of the articles. In the first phase, a reviewer (AH) entered the free and controlled vocabulary in the selected databases and imported them into Covidence. In the second phase, two reviewers (AH and ASV) independently reviewed the titles and abstracts. A 90% level of inter-rater agreement was reached. The conflicts related to the inclusion of the articles were discussed and resolved at every phase of the selection. Studies were eligible for inclusion if they included at least one participant diagnosed with lvPPA, used and analyzed a picture naming task and/or a word repetition task and/or a pseudoword repetition task, and explicitly mentioned the functional origin of the OWP deficit. The detailed inclusion and exclusion criteria are presented in Table 1. Additionally, the review only included peer-reviewed articles to minimize bias in study design. In the third phase, the two independent reviewers (AH and ASV) carried out a full-text screening of the remaining articles. A 92% level of inter-rater agreement was reached. The conflicts relating to the inclusion of the articles were discussed and resolved at every phase of the selection. To extract the data in the fourth phase, one reviewer (AH) read each article and completed a data extraction table with all pertinent information from each of them. A total of three articles from the extracted articles were also reviewed by another author (ASV or LM) to ensure systematicity and coherence. Data relative to general information (authors, title, date of publication, journal of publication, article type and design), general aim of the study, study participants (language, country, number of participants, age range per group, years of education, gender), the cognitive tests used, the manipulated and controlled psycholinguistic properties, the type of errors made by participants, the functional origin of the OWP deficits, and the limits and perspectives of the study were extracted in an Excel document.

### 2.3. Methodological Quality

To ascertain the methodological quality of each study, two reviewers (AH and LM) independently scored each article using the JBI Critical Appraisal Tool [16] by answering yes/no for each given criterion and calculating the total score by adding the number of “yes” answers. The following categories were evaluated: (item 1) a precise and detailed description of the inclusion and exclusion criteria for the sample (e.g., stage of disease progression); (item 2) a precise and detailed description of the study sample (e.g., demographics); (item 3) a valid and reliable method of measuring exposure (e.g., the use of normative, reliable and validated tests to assess OWP); (item 4) objective and standardized criteria for measuring the condition (e.g.,: the use of a specific diagnosis or definition for the evaluated condition); (item 5) identification of cofounding factors (i.e., the presence of factors that could influence the results reported by the authors); (item 6) strategies to adjust cofounding factors (i.e., if some cofounding factors were mentioned, the authors must specify how they included them in their analysis); (item 7) measurement of outcomes in a valid and reliable way (e.g., the assessment of naming in a valid and reliable way); and (item 8) an appropriate statistical analysis (i.e., the presence of a detailed description of the statistical analysis to determine whether the results of the study were properly analyzed and interpreted) for each included article. Each item on the checklist was assessed for all included articles. The conflicts related to the methodological evaluation were discussed between the two reviewers and a consensus was reached for each article.

## 3. Results

### 3.1. Study Selection

**Figure 1:**
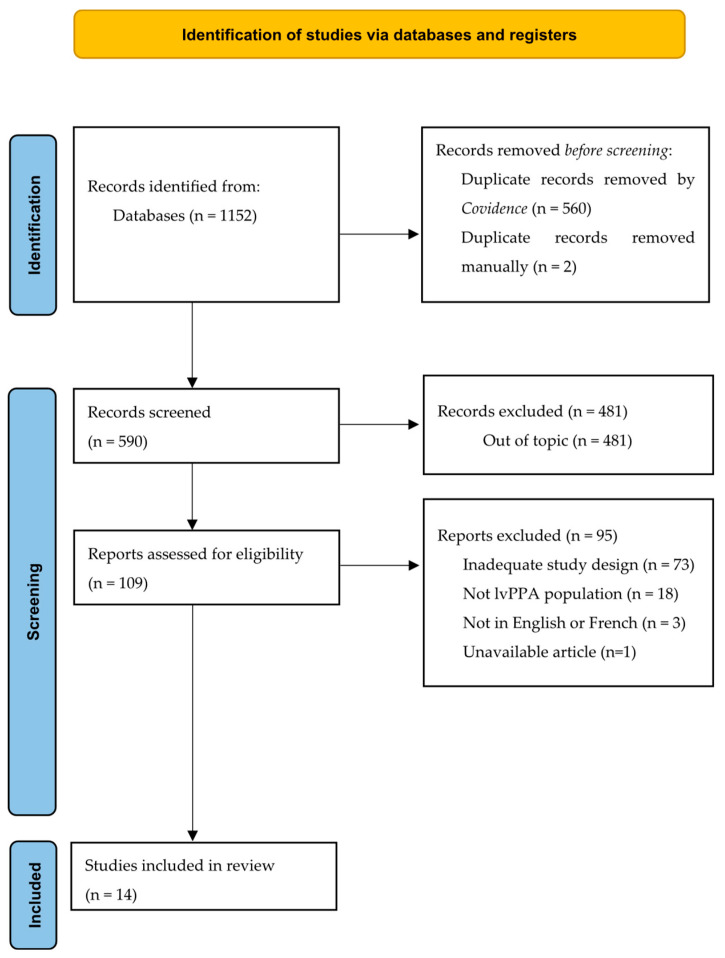
PRISMA flow diagram for study selection.

The initial search yielded 590 studies, after the removal of duplicates. Based on the selection criteria, the screening of the articles’ titles and abstracts led to the exclusion of 481 of them, while 94 other articles were excluded after full-text reading. Therefore, a total of 14 articles were included in this review. Figure 1 presents a PRISMA flowchart synthesizing the article selection process.

### 3.2. Quality Assessment

The methodological quality of the individual studies is shown in Table 2. The results show that most of the articles have a good overall methodological quality (all articles have a score between 6 and 8). However, the criteria that contributed to a lower score were sometimes a missing description of inclusion and exclusion criteria (item 1), a missing identification of confounding factors (item 6), and missing strategies for adjusting for confounding factors (item 7).

### 3.3. General Overview of lvPPA Participants in the Included Studies

Table 3 summarizes the sociodemographic characteristics of the lvPPA participants included in this review. All studies compared lvPPA participants’ results in picture naming tasks and/or repetition tasks to the performance of healthy controls. In all but one of the studies, the lvPPA participants were part of a single experimental group, whereas in the study by Santi and colleagues (2024), they were distributed in two experimental groups, namely lvPPA and lvPPA+ [26]. Some studies also included other types of neurodegenerative diseases or health conditions in their investigation (svPPA, nfvPPA, mixed PPA, different variants of Alzheimer’s disease, Lewy body dementia, progressive supranuclear palsy, corticobasal syndrome, behavioral variant of frontotemporal dementia, and post-stroke), but only participants with lvPPA were included in this systematic review.

### 3.4. Functional Origin of OWP Deficits

The assessment tasks used to identify the functional origin of OWP deficits varied between the included studies. The results are presented in Table 4, including information about the type of task used, the number of stimuli, the manipulation of psycholinguistic properties, and the functional origin of OWP deficits.

## 4. Discussion

The aim of this quantitative systematic review was to synthesize and analyze the literature on the functional origin of OWP deficits in lvPPA. The overall findings of this review suggest that OWP deficits in lvPPA are mainly due to two functional origins: a lexical impairment affecting lexical processing and a post-lexical impairment affecting phonological short-term memory. However, three studies have also suggested the idea of a third functional origin, which concerns the semantic processing level. The following sections discuss each of these three functional origins, considering the tasks used to identify them, the psycholinguistic properties manipulated and/or controlled, and the qualitative error analyses.

### 4.1. Impairment at the Lexical Level

A lexical origin of OWP deficits in lvPPA was identified in nine studies [5,8,18,19,21,22,25,26,27]. These studies employed different versions of picture naming tasks, administered either in isolation or in combination with other tasks. The authors based their conclusions on several factors, including the performance of lvPPA participants compared to healthy controls, the qualitative analyses of errors, and in some cases the effect of psycholinguistic properties of the stimuli, such as lexical frequency and age of acquisition (AoA), which are known to influence lexical access [28,29].

Only in two studies [18,27] did the authors manipulate the psycholinguistic properties of the stimuli and reported effects, which supported their analyses about the lexical functional origin of OWP deficits in lvPPA. Teichmann et al. (2013) showed that lvPPA participants presented abnormal performance on low-frequency words compared to high-frequency words [18]. Interestingly, 68% of the sample (*n* = 12) also presented difficulties with high-frequency words, which potentially diminishes the significance of the reported effects. The lexical frequency of the stimuli was also manipulated by Jebahi et al. (2024), who found a significant effect of this parameter in a picture naming task, though only in three of their fourteen participants with lvPPA [27]. The lack of a clear effect of lexical frequency, a parameter closely linked to lexical access, somewhat calls into question the lexical origin of the impairment suggested by the authors. In their study, Jebahi et al. (2024) also manipulated the AoA of words, another parameter known to influence lexical access, as words acquired earlier in life are known to be more easily retrieved in picture naming [27]. The authors reported that AoA was the psycholinguistic property that most strongly influenced participants’ performance on the picture naming task, both at the individual and the group level, and predicted the naming accuracy for ten participants among fourteen. They concluded that this finding supports the idea that the breakdown occurs at the post-semantic level of phonological processing [27].

The qualitative analysis of the errors produced by lvPPA participants in picture naming tasks was the key element supporting the authors’ conclusion that a lexical impairment underlies the OWP deficit [5,8,18,19,21,22,25,26]. In general, studies identified different types of errors produced by lvPPA patients that could reflect lexical impairment, including omissions [5,8,18,21,22,25], semantic paraphasias [8,22,26], phonological paraphasias [5,8,18,19,21,22], and circumlocutions [5,8,21]. However, according to theoretical models of OWP (e.g., [7]) some of these errors might reflect a semantic rather than a lexical deficit. In one of the reviewed studies, the authors proposed that circumlocutions either served as a compensatory strategy for a lexical access deficit [8] or were an adequate description of the item, also indicating a lexical access deficit [5,21]. Although these interpretations are plausible, a more precise classification into “vague” (e.g., cat: a small animal) or “precise” (e.g., cat: a pet that hunts mice) circumlocutions would provide stronger support for the lexical deficit hypothesis, since only precise circumlocutions typically indicate an impairment of lexical access [[30], while vague circumlocutions typically indicate a semantic impairment. In the studies examined, however, the authors considered circumlocutions as a whole and did not differentiate between the two types. In turn, Budd et al. (2010) suggested that the production of semantic errors in their participants, such as co-ordinate semantic paraphasias (e.g., dog named as ‘’cat’’) and associative semantic paraphasias (e.g., paper named as ‘’pencil’’) might have resulted from an impaired lexical access, as lvPPA participants generally have a relatively well-preserved semantic system [8]. Although this hypothesis is plausible, it should be further substantiated by a comprehensive assessment of the semantic system to demonstrate its integrity or impairment. Semantic errors in picture naming were also reported in the study of Catricala et al. (2020) [22]. However, the authors interpreted these errors as either stemming from an impairment in the retrieval of phonological word forms, at the post-semantic stage, or from a semantic impairment. The hypothesis of a lexical deficit in lvPPA was further supported by Nelson et al. (2023), who examined eye-tracking during a word-to-picture matching task using common objects that lvPPA participants were either able to name correctly or not at all (omissions) in a previous picture naming task [25]. Their performance on correctly and incorrectly named items was similar to that of the control group, suggesting that the omissions were caused by an impairment that was functionally localized at the lexical level, while semantic processing was largely preserved [25]. Once again, this hypothesis should be reinforced by a more controlled evaluation of the semantic system.

In sum, a lexical deficit, reflecting impaired lexical processing, has been identified in the lvPPA population. However, further studies are needed to support this conclusion, as many studies have not considered all the components involved in OWP, such as the influence of psycholinguistic variables known to influence lexical processing (word frequency, AoA), as well as the qualitative analysis of errors.

### 4.2. Impairment at the Post-Lexical Level

A functional origin at the post-lexical stage of OWP, more specifically at the phonological short-term memory, was reported in nine of the included studies [5,9,17,19,20,21,23,24,26].

In eight of them, the identification of a post-lexical impairment was based on the performance of lvPPA participants in word and/or pseudoword repetition tasks [5,9,17,19,20,21,24,26].

The manipulation of specific psycholinguistic properties, such as length, is known to influence phonological short-term memory, hence shorter stimuli are typically better named or repeated than longer ones [31]. In two studies [9,20], the authors found a length effect in an immediate pseudoword repetition task in which performance decreased as the length of the stimuli increased. This can be explained by the fact that performance on a pseudoword repetition task relies heavily on phonological short-term memory, as participants cannot rely on the semantic system or the phonological lexicon to perform the task. Moreover, Meyer et al. (2015) found that the lvPPA participants performed significantly worse on the pseudoword task than the other two groups in the study (controls and participants with Alzheimer’s disease), regardless of the length of the stimuli [20]. Furthermore, Macoir et al. (2024) also found a length effect in the immediate repetition of words with a deficit for 5-syllable words as well as in delayed (5 s) conditions of word and pseudoword repetition [9]. The innovative use of delayed word and pseudoword repetition tasks allows the assessment of phonological short-term memory in a different light, as the information tends to decay rapidly within this component. The length effect found by Macoir et al. (2024) suggests that the deficit likely reflected an impairment at the post-lexical level, specifically within the phonological short-term memory [9].

A total of five studies, that suggested a functional origin at the post-lexical level to explain OWP deficits, based their conclusions on the qualitative analysis of the errors produced by lvPPA participants [5,17,19,24,26]. In all five studies, the presence of phonological errors was interpreted as indicative of a post-lexical deficit, suggesting that the storage capacity of phonological short-term memory was reached and had begun to decline. Santi and colleagues (2024) highlighted that phonological errors were produced by some participants in the immediate pseudoword repetition task in the two lvPPA profiles (9 lvPPA and 18 lvPPA+) reflecting a post-lexical impairment [26]. Similarly, only some participants in Leyton et al. (2015) made significantly more phonological errors in single-word repetition than the other participants [5]. It is important to note that phonological errors can be difficult to categorize, as they can either stem from a lexical or a post-lexical deficit. Therefore, additional assessment tasks are crucially needed to clarify the nature of the OWP deficit, using for example a digit span or word span test to confirm the phonological short-term memory deficit.

In addition to a phonological short-term memory deficit, 4 of the 14 participants in Croot et al. (2012) showed difficulties associated with apraxia of speech in a word repetition task, suggesting impairment at the articulatory level [17]. This result is surprising, as the diagnostic criteria for lvPPA explicitly state that grammatical processing and motor speech need to be unimpaired [2]. However, Croot and colleagues pointed out that phonological and apraxic errors are not always easy to distinguish from one another [17]. Therefore, their presence in PPA variants may not be as distinctly evident in the clinic as with the current clinical criteria [17].

Complementary tasks and methods were also used by the researchers who concluded a post-lexical deficit. A few studies used sentence repetition tasks [9,19,20,24], connected speech [17], forward and backward digit span [19,20], word span [19], and pseudoword and word reading [20], as well as letter and category fluency tasks [23]. 

In summary, a post-lexical deficit that is functionally localized within the phonological short-term memory seems to be distinctly identified in this population.

### 4.3. Impairment at the Semantic Level

Considerable heterogeneity in the clinical presentation of lvPPA has been documented, with some patients exhibiting semantic deficits [32]. Four studies in this review presented lvPPA participants with tasks that specifically assess the semantic system without requiring OWP, such as single-word comprehension [5,19,21,26] and semantic association [5,21,26]. Only one study reported normal performance on single-word comprehension in this population across all participants [21], consistent with current clinical guidelines [2], while others reported deficits in some participants. In these studies, a possible semantic deficit was reported in certain participants, sometimes in combination with a lexical deficit and sometimes with a post-lexical deficit, and it was identified as a partial explanation for OWP deficits in individuals with lvPPA. In addition, participants in the study of Santi and colleagues (2024) were divided into two subgroups, both exhibiting predominant anomia and sentence repetition impairments [26]. However, one subgroup showed additional mild semantic deficits characterized by errors in naming, semantic association, and single-word comprehension tasks (referred to as lvPPA+), while the other subgroup showed no semantic deficits (referred to as lvPPA). The authors pointed out that the use of the classification “lvPPA+” remains uncertain given its recent introduction. Of note, participants in the lvPPA+ subgroup not only showed semantic deficits but also a longer duration of symptoms compared to the lvPPA group. The semantic impairments could therefore reflect either an atypical presentation of lvPPA or the progression of the lvPPA condition over time. [26]

The qualitative analysis of the errors produced on a picture naming task, by the lvPPA participants of the three studies reporting semantic deficits, was used to explain, at least partially, the OWP deficits observed in the latter. In both studies by Leyton et al., the participants who showed semantic impairments in the semantic tasks made coordinate and superordinate substitution errors (e.g., lion named as ‘’animal’’), which were categorized as “semantic errors”. They also produced circumlocutions [5,21]. However, coordinate errors may also result from lexical access difficulties, and circumlocutions may be due to a semantic or lexical impairment. Thus, it could be relevant to examine more closely the errors reported (e.g., classify circumlocutions into ‘’vague’’ or “precise’’) to determine which component(s) is impaired, and to manipulate psycholinguistic properties such as frequency to better characterize the lexical system and familiarity to better categorize the semantic system. Furthermore, Santi et al. (2024) confirmed the presence of semantic errors in picture naming tasks in some of their lvPPA+ participants, without specifying their nature [26].

The activation of conceptual representations in semantic memory is an integral part of the processes of OWP. Therefore, an impairment functionally localized in the semantic system will have a negative impact on performance in OWP tasks. Consequently, assessing the integrity of the semantic component using tasks that do not require OWP (e.g., semantic questionnaires, written word semantic matching) is essential to adequately identify the functional origin of OWP impairments in all clinical populations, including lvPPA. This should include the manipulation of psycholinguistic variables (i.e., concept familiarity, semantic category), which are known to influence semantic processing as well as the interpretation of errors (e.g., vague vs. precise circumlocutions).

In sum, a certain heterogeneity in the clinical profile of lvPPA has been reported in the literature [10]. In three studies of this review, some participants with lvPPA showed semantic impairments on various assessment tasks, including those recruiting the OWP abilities. However, this impairment was always combined with deficits that were functionally localized at the lexical or post-lexical level. The semantic origin of the OWP deficits thus seems to be related to an atypical rather than a typical presentation of the lvPPA.

### 4.4. Limitations and Future Directions

The decision to include only studies in which the researchers explicitly stated the functional origin of OWP, in order to avoid possible misinterpretation of their results, is a potential limitation of this systematic review. Due to this methodological decision, studies that could provide valuable indirect insights, such as intervention studies, might have been excluded. In addition, the protocol of the present review was not registered in a public database such as PROSPERO.

Despite the overall good methodological quality of the selected studies, the lack of description of the inclusion and exclusion criteria in some of them and the unidentified confounding factors in half of the selected studies limit the interpretation of the results and the conclusions drawn. It will be important to consider these methodological criteria in future studies in order to reduce possible bias and misinterpretation.

This systematic literature review provides an overview of the current literature on the functional origin of OWP in the lvPPA. However, there are still some unknowns, particularly in relation to the presence of semantic disorders associated with lexical and/or post-lexical deficits in lvPPA, at least in certain patients. Further clinical studies are also needed to chart the progression and the functional origins of OWP deficits over the course of the disease. A combination of methodological approaches like behavioral assessments with neuroimaging, could significantly improve the understanding of the functional origin of OWP deficits in the lvPPA population.

## 5. Conclusions

This quantitative systematic review of the literature has shown that OWP deficits in lvPPA can have two main functional origins, namely a deficit that impairs the retrieval of phonological representations in the output phonological lexicon and/or a post-lexical deficit that impairs the retention of information in phonological short-term memory. In a few patients, these deficits are combined with an impairment of semantic processing.

This study has also shown that the nature of the assessment tasks, the qualitative analysis of errors, and the manipulation of the psycholinguistic properties of verbal stimuli can partially explain the heterogeneity found in the functional origins of OWP deficits in lvPPA. A standardization of methods and a better consideration of assessment principles seem to be necessary. Indeed, the manipulation of more psycholinguistic properties such as concreteness, familiarity, or word imageability and more accurate error analysis are needed to draw solid conclusions about the OWP deficits observed in the lvPPA population and to determine more precisely the degree of impairment(s).

## Figures and Tables

**Table 1:** Inclusion and exclusion criteria.

Inclusion Criteria	Exclusion Criteria
1. Published in English or French 2. Empirical studies presenting original data 3. Includes at least one participant with a diagnosis of lvPPA, confirmed by neuroimaging, biomarkers, and/or Gorno-Tempini et al. criteria [2] 4. Includes an impaired picture naming and/or word repetition and/or pseudoword repetition task 5. Mentions explicitly the functional origin of the oral word production deficit 6. Peer reviewed	1. No distinction between the lvPPA from other PPA variants or neurodegenerative disorders 2. No description of the picture naming and/or word repetition and/or non-word repetition task 3. Treatments and intervention programs

**Table 2:** Methodological Evaluation of the Selected Studies using the JBI Critical Appraisal Tool.

Article	Item 1	Item 2	Item 3	Item 4	Item 5	Item 6	Item 7	Item 8	Total
[8] (Budd et al., 2010)	Yes	Yes	Yes	Yes	Yes	Yes	Yes	Yes	8
[17] (Croot et al., 2012)	Yes	Yes	Yes	Yes	No	No	Yes	Yes	6
[18] (Teichmann et al., 2013)	Yes	Yes	Yes	Yes	Yes	Yes	Yes	Yes	8
[19] (Leyton et al., 2014)	Yes	Yes	Yes	Yes	No	No	Yes	Yes	6
[20] (Meyer et al., 2015)	No	Yes	Yes	Yes	Yes	Yes	Yes	Yes	7
[5] (Leyton et al., 2015)	Yes	Yes	Yes	Yes	Yes	Yes	Yes	Yes	8
[21] (Leyton et al., 2017)	Yes	Yes	Yes	Yes	Yes	Yes	Yes	Yes	8
[22] (Catricala et al., 2020)	Yes	Yes	Yes	Yes	No	No	Yes	Yes	6
[23] (Putcha et al., 2020)	Yes	Yes	Yes	Yes	Yes	No	No	Yes	6
[24] (Macoir et al., 2021)	Yes	Yes	Yes	Yes	Yes	No	No	Yes	6
[25] (Nelson et al., 2023)	No	Yes	Yes	Yes	Yes	Yes	Yes	Yes	7
[26] (Santi et al., 2024)	Yes	Yes	Yes	Yes	Yes	Yes	Yes	Yes	8
[9] (Macoir et al., 2024)	Yes	Yes	Yes	Yes	No	No	Yes	Yes	6
[27] (Jebahi et al., 2024)	Yes	Yes	Yes	Yes	No	No	Yes	Yes	6

**Table 3:** Sociodemographic Characteristics of the Participants in the Selected Studies and the Methodological Evaluation.

Reference	Participant Characteristics					
	N (lvPPA)	Diagnostic Method	Mean Age (SD)	Men: Women	Education, Years (SD)	Duration, Mean Years After Diagnosis (SD)
[8] (Budd et al., 2010)	13	Clinical guidelines + MRI scans or SPECT or PET	69 (8.2)	8:5	15.8 (2.9)	-
[17] (Croot et al., 2012)	14	Clinical guidelines	66.2	5:9	14.4	3.6
[18] (Teichmann et al., 2013)	19	Clinical guidelines	66.5 (8.7)	13:6	11.8 (3.8)	3.2 (0.6)
[19] (Leyton et al., 2014)	10	Clinical protocol + Clinical guidelines	64.5 (8.2)	4:6	13.0 (3.2)	4.6
[20] (Meyer et al., 2015)	11	Clinical guidelines	70.7(8.3)	4:7	17.3 (1.6)	-
[5] (Leyton et al., 2015)	21	Clinical protocol + Clinical guidelines	66.9 (7.6)	7:14	13.2 (3.6)	3.5 (2.2)
[21] (Leyton et al., 2017)	22	Clinical guidelines	67.6 (8.3)	10:12	12.3 (3.2)	4.0 (2.8)
[22] (Catricala et al., 2020)	28	Clinical guidelines + FDG-PET, CSF, amyloid PET	69.57(6.98)	15:13	10.6 (2.18)	-
[23] (Putcha et al., 2020)	22	Clinical guidelines + CSF + amyloid PET	69.4 (7.1)	15:7	16.4(2.5)	-
[24] (Macoir et al., 2021)	4	Clinical guidelines + FDG-PET scan	66.75 (8.14)	2:2	14.25 (5.25)	2.5
[25] (Nelson et al., 2023)	12	Clinical guidelines	67.4 (2.5)	9:3	15.5 (0.7)	5.3 (0.7)
[26] (Santi et al., 2024)	19 lvPPA	Clinical guidelines	68.37 (6.13) lvPPA	10:9	11.11 (3.19)	1.8
	23 lvPPA+		71.74 (7.53) lvPPA+	10:13	11.70 (4.80)	2.8
[9] (Macoir et al., 2024)	11	Clinical guidelines + CT scan + PET scan + lumbar puncture	67.36 (6.45)	6:5	13.36 (2.54)	4 (2.19)
[27] (Jebahi et al., 2024)	14	Clinical guidelines	70.14 (6.68)	3:11	17.43 (4.60)	3.17 (1.69)

**Table 4:** Functional Origin of Oral Word Production Deficits in the Selected Studies.

Article	Type of Task	Number of Stimuli	Psycholinguistic Properties	Functional Origin
[8] (Budd et al., 2010)	Picture Naming	30	N/A	Lexical deficit: Access to phonological representations
[22] (Catricala et al., 2020)	Picture Naming	48	N/A	Lexical deficit: Retrieval of phonological form
[23] (Putcha et al., 2020)	Picture naming	30	N/A	Post-lexical deficit: Phonological loop
[25] (Nelson et al., 2023)	Picture naming	60	N/A	Lexical deficit: lexical access deficit or phonological encoding deficit
[18] (Teichmann et al., 2013)	Picture Naming	80	Frequency	Lexical deficit: Impaired access to lexical representations Lexical deficit: Output lexicon
[27] (Jebahi et al., 2024)	Picture Naming	60	Familiarity, Frequency, Age of acquisition, Length, Phonological neighborhood density, Semantic neighborhood density, Arousal, Valence	Lexical deficit: Post-semantic phonological processing level
[17] (Croot et al., 2012)	Word repetition	30	N/A	Post-lexical deficit: Phonological short-term memory deficit
[20] (Meyer et al., 2015)	Word and pseudoword repetition	10 words 30 pseudowords	Length	Post-lexical deficit: Phonological short-term memory
[24] (Macoir et al., 2021)	Word and pseudoword repetition	10 words 10 pseudowords	Length, Syllable structure	Post-lexical deficit: Phonological short-term memory
[9] (Macoir et al., 2024)	Immediate and delayed word and pseudoword repetition	50 words 50 pseudowords	Length, Lexicality	Post-lexical deficit: Phonological short-term memory deficit
[19] (Leyton et al., 2014)	Picture naming	30	N/A	Lexical deficit: Phonological output Post-lexical deficit: Phonological input buffer
	Word repetition	30	N/A	
[5] (Leyton et al., 2015)	Picture naming	30	Category	Lexical deficit: Pure anomia (lexical access) Lexical deficit + Semantic deficit: lexical access + semantic processing impairment Lexical + post-lexical deficit: Retrieving phonological form of words + phonological output processing
	Word repetition	30	N/A	
[21] (Leyton et al., 2017)	Picture naming	30	N/A	Lexical-Semantic deficit: Semantic + phonological impairment Post-lexical deficit: phonological processing
	Word repetition	30	N/A	
[26] (Santi et al., 2024)	Picture naming	14	N/A	Lexical deficit Lexical deficit + semantic deficit Lexical deficit + semantic + post-lexical deficit
	Word and pseudoword repetition	6 words, 4 pseudowords	N/A	

## Data Availability

No new data were created or analyzed in this study. Data sharing is not applicable to this article.

## Supplementary Materials

The following supporting information can be downloaded at: https://www.mdpi.com/article/10.3390/brainsci15020111/s1, Terminology and Search Terms.

## References

[1] Mesulam M.-M., Rogalski E.J., Wieneke C., Hurley R.S., Geula C., Bigio E.H., Thompson C.K., Weintraub S. (2014). Primary Progressive Aphasia and the Evolving Neurology of the Language Network. Nat. Rev. Neurol..

[2] Gorno-Tempini M.L., Hillis A.E., Weintraub S., Kertesz A., Mendez M., Cappa S.F., Ogar J.M., Rohrer J.D., Black S., Boeve B.F. (2011). Classification of Primary Progressive Aphasia and Its Variants. Neurology.

[3] Meyer A.M., Snider S.F., Tippett D.C., Saloma R., Faria A.V., Turkeltaub P.E., Hillis A.E., Friedman R.B. (2024). The Pattern of Phonological, Semantic, and Circumlocution Naming Errors for Nouns and Verbs in Primary Progressive Aphasia. Aphasiology.

[4] Gorno-Tempini M.L., Dronkers N.F., Rankin K.P., Ogar J.M., Phengrasamy L., Rosen H.J., Johnson J.K., Weiner M.W., Miller B.L. (2004). Cognition and Anatomy in Three Variants of Primary Progressive Aphasia. Ann. Neurol..

[5] Leyton C.E., Hodges J.R., McLean C.A., Kril J.J., Piguet O., Ballard K.J. (2015). Is the Logopenic-Variant of Primary Progressive Aphasia a Unitary Disorder?. Cortex.

[6] Dell G.S., Schwartz M.F., Martin N., Saffran E.M., Gagnon D.A. (1997). Lexical Access in Aphasic and Nonaphasic Speakers. Psychol. Rev..

[7] Levelt W.J.M. (1992). Accessing Words in Speech Production: Stages, Processes and Representations. Cognition.

[8] Budd M.A., Kortte K., Cloutman L., Newhart M., Gottesman R.F., Davis C., Heidler-Gary J., Seay M.W., Hillis A.E. (2010). The Nature of Naming Errors in Primary Progressive Aphasia versus Acute Post-Stroke Aphasia. Neuropsychology.

[9] Macoir J., Laforce R., Lavoie M. (2024). The Impact of Phonological Short-Term Memory Impairment on Verbal Repetition in the Logopenic Variant of Primary Progressive Aphasia. Neuropsychol. Dev. Cogn. Sect. B Aging Neuropsychol. Cogn..

[10] Conca F., Esposito V., Giusto G., Cappa S.F., Catricalà E. (2022). Characterization of the Logopenic Variant of Primary Progressive Aphasia: A Systematic Review and Meta-Analysis. Ageing Res. Rev..

[11] Liuzzi A.G., Meersmans K., Peeters R., De Deyne S., Dupont P., Vandenberghe R. (2024). Semantic Representations in Inferior Frontal and Lateral Temporal Cortex during Picture Naming, Reading, and Repetition. Hum. Brain Mapp..

[12] Grima R., Franklin S. (2017). Usefulness of Investigating Error Profiles in Diagnosis of Naming Impairments. Int. J. Lang. Commun. Disord..

[13] Wilson S.M., Isenberg A.L., Hickok G. (2009). Neural Correlates of Word Production Stages Delineated by Parametric Modulation of Psycholinguistic Variables. Hum. Brain Mapp..

[14] Oldfield R.C., Wingfield A. (1965). Response Latencies in Naming Objects. Q. J. Exp. Psychol..

[15] Page M.J., Moher D., McKenzie J.E. (2022). Introduction to PRISMA 2020 and Implications for Research Synthesis Methodologists. Res. Synth. Methods.

[16] Munn Z., Barker T.H., Moola S., Tufanaru C., Stern C., McArthur A., Stephenson M., Aromataris E. (2020). Methodological Quality of Case Series Studies: An Introduction to the JBI Critical Appraisal Tool. JBI Evid. Synth..

[17] Croot K., Ballard K., Leyton C.E., Hodges J.R. (2012). Apraxia of Speech and Phonological Errors in the Diagnosis of Nonfluent/Agrammatic and Logopenic Variants of Primary Progressive Aphasia. J. Speech Lang. Hear. Res..

[18] Teichmann M., Kas A., Boutet C., Ferrieux S., Nogues M., Samri D., Rogan C., Dormont D., Dubois B., Migliaccio R. (2013). Deciphering Logopenic Primary Progressive Aphasia: A Clinical, Imaging and Biomarker Investigation. Brain.

[19] Leyton C.E., Savage S., Irish M., Schubert S., Piguet O., Ballard K.J., Hodges J.R. (2014). Verbal Repetition in Primary Progressive Aphasia and Alzheimer’s Disease. J. Alzheimers Dis..

[20] Meyer A.M., Snider S.F., Campbell R.E., Friedman R.B. (2015). Phonological Short-Term Memory in Logopenic Variant Primary Progressive Aphasia and Mild Alzheimer’s Disease. Cortex.

[21] Leyton C.E., Hodges J.R., Piguet O., Ballard K.J. (2017). Common and Divergent Neural Correlates of Anomia in Amnestic and Logopenic Presentations of Alzheimer’s Disease. Cortex.

[22] Catricala E., Polito C., Presotto L., Esposito V., Sala A., Conca F., Gasparri C., Berti V., Filippi M., Pupi A. (2020). Neural Correlates of Naming Errors across Different Neurodegenerative Diseases: An FDG-PET Study. Neurology.

[23] Putcha D., Dickerson B.C., Brickhouse M., Johnson K.A., Sperling R.A., Papp K.V. (2020). Word Retrieval across the Biomarker-Confirmed Alzheimer’s Disease Syndromic Spectrum. Neuropsychologia.

[24] Macoir J., Martel-Sauvageau V., Bouvier L., Laforce R., Monetta L. (2021). Heterogeneity of Repetition Abilities in Logopenic Variant Primary Progressive Aphasia. Dement. Neuropsychol..

[25] Nelson M.J., Moeller S., Seckin M., Rogalski E.J., Mesulam M.-M., Hurley R.S. (2023). The Eyes Speak When the Mouth Cannot: Using Eye Movements to Interpret Omissions in Primary Progressive Aphasia. Neuropsychologia.

[26] Santi G.C., Conca F., Esposito V., Polito C., Caminiti S.P., Boccalini C., Morinelli C., Berti V., Mazzeo S., Bessi V. (2024). Heterogeneity and Overlap in the Continuum of Linguistic Profile of Logopenic and Semantic Variants of Primary Progressive Aphasia: A Profile Analysis Based on Multidimensional Scaling Study. Alzheimers Res. Ther..

[27] Jebahi F., Nickels K.V., Kielar A. (2024). Predicting Confrontation Naming in the Logopenic Variant of Primary Progressive Aphasia. Aphasiology.

[28] Juhasz B.J. (2005). Age-of-Acquisition Effects in Word and Picture Identification. Psychol. Bull..

[29] Spieler D.H., Balota D.A. (2000). Factors Influencing Word Naming in Younger and Older Adults. Psychol. Aging.

[30] Nicholas M., Obler L., Albert M., Goodglass H. (1985). Lexical Retrieval in Healthy Aging. Cortex.

[31] Nickels L., Howard D. (1995). Aphasic Naming: What Matters?. Neuropsychologia.

[32] Watanabe H., Hikida S., Ikeda M., Mori E. (2022). Unclassified Fluent Variants of Primary Progressive Aphasia: Distinction from Semantic and Logopenic Variants. Brain Commun..

