# Supplementary material for: The Functional Origin of Oral Word Production Deficits in the Logopenic Variant of Primary Progressive Aphasia: A Systematic Review
Source: Brain Sci. 2025 Jan 24;15(2):111. doi: 10.3390/brainsci15020111 (PMC11853272; doi:10.3390/brainsci15020111)
Supplement: Supplementary file 1 [file brainsci-15-00111-s001.zip › brainsci-3405468-supplementary.pdf]

Supplementary Materials

Medline via OVID
("primary progressive aphasia" or "PPA" or "logopenic" or "lv-PPA" or "PPA-L").ti,ab. or exp Aphasia, Primary Progressive/<br>AND<br>(anomi* or lexical retriev* or word retriev* or "word production" or "word finding" or "naming" or "repetition").ti,ab. OR Anomia/
**PsycINFO via OVID**
("primary progressive aphasia" OR "PPA" OR "logopenic" OR "lv-PPA" OR "PPA-L").ti,ab.<br>AND<br>(anomi* OR lexical retriev* OR word retriev* OR "word production" OR "word finding" OR "naming" OR "repetition").ti,ab. OR Lexical retrieval/
**Linguistics and Language Behavior Abstracts**
(Title ("primary progressive aphasia" OR "PPA" OR "logopenic" OR "lv-PPA" OR "PPA-L") OR Abstract ("primary progressive aphasia" OR "PPA" OR "logopenic" OR "lv-PPA" OR "PPA-L"))<br>AND<br>(Title (anomi* OR lexical retriev* OR word retriev* OR "word production" OR "word finding" OR "naming" OR "repetition") OR Abstract (anomi* OR lexical retriev* OR word retriev* OR "word production" OR "word finding" OR "naming" OR "repetition")) OR MAINSUBJECT.EXACT("Anomia" OR "Lexical Access")<br><br>Filters:<br>-Peer reviewed only<br>-Languages: English, French<br>-Document type: Articles
**CINAHL via EBSCO**
TI ("primary progressive aphasia" OR "PPA" OR "logopenic" OR "lv-PPA" OR "PPA-L") OR AB ("primary progressive aphasia" OR "PPA" OR "logopenic" OR "lv-PPA" OR "PPA-L")<br>AND<br>TI (anomi* OR lexical retriev* OR word retriev* OR "word production" OR "word finding" OR "naming" OR "repetition") OR AB (anomi* OR lexical retriev* OR word retriev* OR "word production" OR "word finding" OR "naming" OR "repetition") OR MH ("anomia")
